# Notes and Notices

**Published:** 1896-05

**Authors:** 


### NOTES AND NOTICES.

THE annual meeting of the SOCIETY OF HOMŒOPATHICIANS will be held at "The Matthewson," Narragansett Pier, R. I., June 23d, 24th, 25th, and 26th.

The rates will be $3.00 per day; two in a room, $5.00 per day.

"The Matthewson" has been much enlarged, almost rebuilt, and will be more attractive than ever.

Four days have been selected for the meeting, in order to avoid the constant work and hurry that usually attends such conventions; it will also give those present a chance to enjoy themselves and to become acquainted with one another.

The meeting will be an excellent one. Many interesting papers are promised for the various bureaus.

Candidates for membership will be present and will read papers.

A stenographer will be in attendance.

The first session will be held at 2.30 P. M., Tuesday June 23d.

S. A. KIMBALL, *Secretary*,
124 Commonwealth Ave.,
Boston, Mass.

"THE PLEASURES OF OUT-DOOR LIFE." Birds, insects, ferns, mosses, plants, flowers, stars, planets, etc., are all delightfully written about by the most talented writers in *The Observer*, Portland, Conn. This popular magazine has been greatly enlarged and improved, yet the price is only ten cents a single copy, one dollar a year, as heretofore. Young people as well as old will be interested in the attractive pages of this magazine. It is a valuable opening to the wonderland of out-door life, which, strange to say, is unnoticed by the greater part of mankind.

THE MEDICAL CENTURY has issued a voluminous prospectus of their *Homœopathic Text Book of Surgery*. It contains portraits of the authors and otherwise gives considerable information concerning their book. Copies may be had by addressing The Medical Century Co., 31 Washington Street, Chicago, Ill.

THE STANDARD DICTIONARY of Funk & Wagnalls, New York. Professor D. G. Brinton, M. D., LL. D., University of Pennsylvania, and President of the American Association for the Advancement of Science: "The attack upon the *Standard Dictionary* is absurd, malicious, and inexcusable. The *Standard Dictionary* was unquestionably right in inserting the class of words referred to, right by the precedent referred to in the most cultivated countries, and right by the requirements of linguistic science."

THE AMERICAN HOMŒOPATHIST has arranged to take a select, private party of ladies and gentlemen to London, sailing July 25th, to attend the International Homœopathic Congress.

The cost of this trip will be as follows: From Detroit to London, and return to Detroit, $90.00; from Cleveland to London, and return to Cleveland, $92.00; from Buffalo to London, and return to Buffalo, $85.00. And so in decreasing proportion as we approach the point of sailing. This includes railway fare to point of sailing, ocean fare, and railway fare from Liverpool to London, and return to point of starting.

This is to be a popular, hard-times outing for hard-worked and not over-paid professional people. A lot of jolly folks out for a jolly time and at moderate expense. No full-dress affair. No purple and fine linen. No trunks. Nothing but a valise or two. Wearing comfortable clothing. Also paper collars and cuffs if we want to. Skipping costly hotels. Eating and drinking (principally the former) when and where and as much or as little as we like or can pay for.

The sailing is along a route not commonly selected by American tourists. It is, however, bright with attractive features, both on land and sea. The actual ocean voyage is cut down to about five days.

By going with us you will be with friends. You will not need to be nervous. You will not be a stranger in a strange land. You will not have to deal with unknown and indifferent steamship companies and their local agents. You will deal directly with the editor of *The American Homœopathist*, who will be of the party. Before you leave home you will know exactly the location of your berth, the amount of clothes and money, etc., to take with you.

Board and bed can be had abroad for $1.00 a day and upwards. Arrangements are now pending for special hotel and furnished-room rates in Liverpool, London, and Paris for this party.

Write quickly for particulars (inclosing stamp), as the number of berths held for this party has already been agreed upon, and no more are to be had on this steamship.

Address *The American Homœopathist*, 57 Bell Ave., Cleveland, Ohio.

International Homœopathic Congress, August 3d to 8th, 1896. In consequence of the demand for a hearing at this Assembly, it has been determined that the forenoons, hitherto destined for extemporized and informal gatherings, shall be utilized for "overflow meetings," to be held under the rules and officers of the Congress. They will be devoted to the further discussion of the subjects of the preceding afternoon or to the handling of fresh subjects of the same order.

As a good deal more time will thus be made available, the officers can abandon the limitations under which, in their "preliminary announcement," they invited further communications. They will now welcome such, not only "on the topics hitherto specified, and on those which will be later announced as chosen by the American Committee," but upon any subject which may be selected by the essayist. They would add, moreover, that even should the additional time prove insufficient for a discussion of all the papers they may receive, these—if accepted—will be read by title at the meeting, and be printed in the Transactions.

All American contributions should be sent to Dr. Dewey, 170 West 54th Street, New York, the Secretary of the Committee appointed at the last meeting of the American Institute of Homœopathy for furthering the interests of the Congress from that side of the water. Contributions from other countries should be addressed to the General Secretary of the Congress, Dr. Hughes, Brighton, England.

BURNETT'S COD LIVER OIL is a standard with the medical profession throughout the whole United States. Write to Theo. Metcalf Co., of Boston, for literature.

THE AMERICAN INSTITUTE OF HOMŒOPATHY will hold its annual meeting at Detroit, Mich., beginning Wednesday, June 17th, 1896. The local committee, Dr. D. A. MacLachlan, Chairman, has been vigorously at work during the past few months, and has perfected its plans to such a degree that it may be said, without any exaggeration, that the Institute will receive a right royal welcome in Detroit. A magnificent building, containing auditoriums, large and small, reception rooms, rooms for committees and officers, and every possible convenience, has been engaged for the use of the Institute, and it is believed that the arrangements in this respect will be more complete and satisfactory than ever before. The hotels are first-class, charge moderate prices, and will do all that is possible to entertain the members of the Institute.

Detroit is a beautiful city, centrally located, and most fortunate in its approaches. From it many delightful trips and excursions may be taken. The details of these will be announced by the local committee. One proposed trip, however, deserves special mention—the journey by the magnificent new lake steamers to Duluth and return. There is no finer trip than this in the world.

The annual circular, to be issued in May, will give full information regarding the details of the meeting.

DR. EDMUND CARLETON has removed from 53 West Forty-fifth Street to 62 West Forty-ninth Street.

HAHNEMANN'S BIRTHDAY.—Dr. Henry M. Smith, 288 St. Nicholas Ave., New York City, Secretary and Treasurer of Hahnemann Monument Committee, has written a letter to the profession repeating the offer of Dr. Bushrod W. James, of Philadelphia, Pa., that the birthday of Hahnemann be made the occasion of interesting the public, as well as the profession, in Hahnemann's history. He also reports the great work already accomplished in attaining the object, and solicits more subscriptions, which may be sent to the above address.

MARCHAND'S GLYCOZONE —Recently a case of imitation of this preparation which has been so extensively advertised in THE HOMŒOPATHIC PHYSICIAN, for the past two or three years, has been brought to the attention of the profession.

For some months a Dr. Beach has been selling a similar preparation, which he called Glycozene upon the labels of the vials, but Glycozone in his circulars, thus making it appear to be the same preparation as that of the Drevet Manufacturing Company. This company, therefore, took the case to the United States Circuit Court of Northern Ohio, and obtained an injunction forbidding the sale of the imitation or of the use of the name Glycozone. It is likely that this decision of the court will intimidate others from similar attempts to appropriate these names or the products.

The Pennsylvania State College, State College, Pa.—An examination of candidates for admission will be held at the College, Thursday, June 18th, at 9 o'clock A. M. A second examination will be held Tuesday, September 15th, beginning at the same hour.

Local examinations will also be held Wednesday, June 24th, at Philadelphia, Pittsburg, Harrisburg, Williamsport, Reading, and Scranton, beginning at 9 o'clock A. M. Places will be announced in the local papers two weeks in advance.

Orders for tickets over the Pennsylvania Railroad and branches to Lemont, or to the College (via Bellefonte) and over the Reading and Beech Creek roads to Bellefonte, may be obtained from John I. Thompson, Jr., State College, Pa.

The National Eclectic Medical Association will hold its twenty-sixth annual meeting, by special invitation, in the Chamber of Commerce Building, Portland, Oregon, on the 16th, 17th, and 18th of June, 1896. The Association has issued a handsome octavo book of more than ninety pages, describing the route and giving programme of proceedings. It is embellished with photographs of the fine scenery along the route. The book may be had of the publisher, Pitts Edwin Howes, M. D., Boston, Mass.

### A New Sanatorium.

Lake Avenue, Newton Highlands, Mass., May 12th, 1896.

Dear Doctor:—Every physician occasionally wishes to send away, for a few weeks or months, a patient who needs a change of scene and a rest from business or family cares. To those of my professional friends who hesitate to send such a case to a large sanatorium, and are unwilling to risk a change of treatment, I desire to announce that I am prepared to receive a few cases into my own home and give them careful nursing, a liberal diet, and strictly Homœopathic treatment; avoiding the factitious aid of tonics and palliatives.

Newton Highlands is nine miles from Boston, on the Albany road. The well-known beauty and healthfulness of this suburb render it especially desirable for all who appreciate attractive scenery, pure air and water, fine roads, and proximity to the Atlantic seaboard.

My house is pleasant and modern, located in the healthiest part of Newton. The terms are $25 a week, including board, nursing, and medical attendance.

Yours fraternally,

Samuel L. Eaton, M. D.

